# Supplementary material for: The interactome of intact mitochondria by cross-linking mass spectrometry provides evidence for coexisting respiratory supercomplexes
Source: Mol Cell Proteomics. 2017 Dec 8;17(2):216–32. doi: 10.1074/mcp.RA117.000470 (PMC5795388; doi:10.1074/mcp.RA117.000470)
Supplement: Supplemental Data [file supp_RA117.000470_133922_0_supp_23978_fzffwf.zip › spectra_annotation/mito_spectra_annotation/349-1-2-1-4-1.pdf]

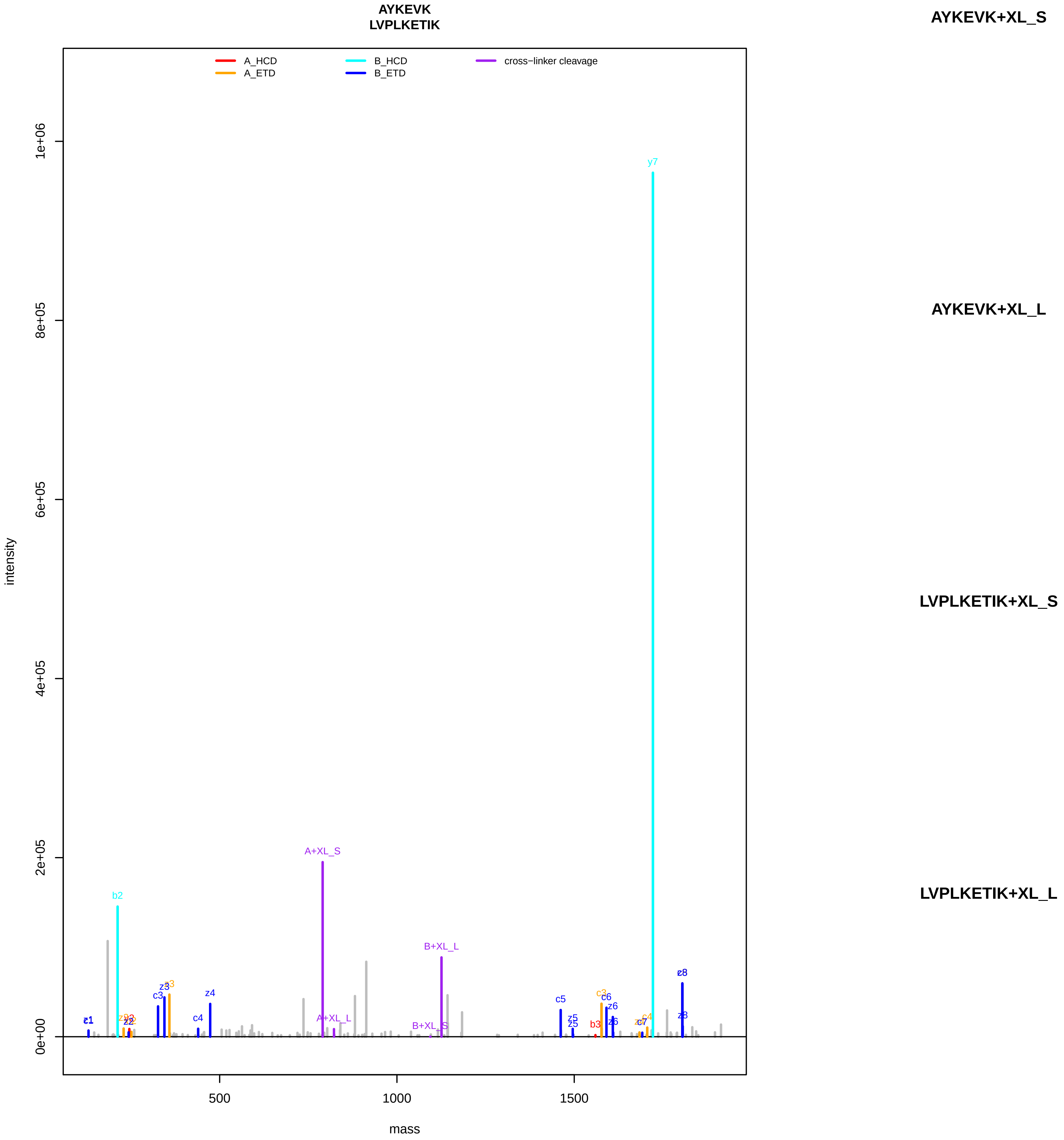
AYKEVK
LVPLKETIK
AYKEVK+XL_S
AYKEVK+XL_L
LVPLKETIK+XL_S
LVPLKETIK+XL_L
A_HCD
A_ETD
B_HCD
B_ETD
cross−linker cleavage
y7
A+XL_S
b2
B+XL_L
z8
c3
c6
z6
c5
z4
z3
c3
c4
A+XL_L
B+XL_S
z5
b3
c7
c4
intensity
mass
0e+00
2e+05
4e+05
6e+05
8e+05
1e+06
500
1000
1500
